# Knowledge of and support for cancer patient pathways among general practitioners and other physicians – a study from Sweden

**DOI:** 10.1080/02813432.2021.1880074

**Published:** 2021-02-08

**Authors:** Christian Backman, Ulf Johansson, Mikko Hellgren

**Affiliations:** a School of Medical Sciences, Örebro University, Örebro, Sweden; b University Health Care Research Center, Örebro University Hospital, Örebro, Sweden

**Keywords:** Cancer patient pathway, primary health care, cancer care pathway, cancer, general practitioner

## Abstract

**Objective:**

To investigate the expertise in and support of the implemented new method of cancer patient pathways (CPPs) among general practitioners (GPs) and other working physicians in Sweden.

**Design:**

A survey in the form of 10 knowledge-based multiple-choice questions (MCQs) and two general questions about CPPs.

**Setting:**

Physicians from two different regions in Sweden answered the survey between December 2018 and January 2019.

**Subjects:**

GPs in primary care compared to other physicians. 155 participants completed the survey and the response rate was 65%.

**Main outcome measures:**

Physicians’ self-estimated knowledge of CPPs in general and opinion of CPPs effect on mortality and morbidity. Their scores on 10 different MCQs. Scores were analysed in subgroups related to the physicians medical specialty and experience.

**Results:**

A majority of all physicians (63%) felt that they had insufficient knowledge regarding the procedure of CPPs, and the average score from the MCQs was 3.8 out of 10 correct answers. The results showed that GPs performed significantly better than specialists from other disciplines.

**Conclusions:**

The low percentage of correctly answered MCQs shows that the information about the entry part of CPPs needs to be improved. The study demonstrates a support for the system with CPPs because the physicians believed in its’ positive effects on morbidity and mortality, however, it also reveals a lack of self-estimated knowledge about the system with CPPs.

**Key points**
- Cancer patient pathways (CPPs) is a newly implemented method in Sweden that aims to equalize cancer care and reduce the time to diagnosis and treatment.
- The proficiency of when to initiate an investigation according to a specific CPP seems low. General practitioners (GPs) performed significantly better on knowledge-based questions than other specialists did.
- Physicians rated their knowledge as insufficient regarding the procedure of CPPs.
- A clear majority of physicians believed that CPPs promotes a lower mortality and morbidity in cancer.

## Introduction

Cancer continues to be one of the leading causes of death in both low- and high-income countries worldwide [1,2]. In Europe, the eastern European countries have the highest cancer burden, while the lowest is found in Scandinavia (except Denmark) [3]. To improve the cancer survivability rates, different countries in Europe have initiated new national strategies to reduce diagnostic delays such as a cancer patient pathways (CPPs) in Denmark or a scheme for 2-week referral after cancer suspicion in England [4,5].

The start of a diagnostic process in a physician–patient consultation involves different characteristics. To put it simply, the diagnostic process starts in the meeting with a patient, during which the physician uses his/her ability for pattern recognition, hypothesis-driven examinations, clinical experience and intuition to narrow down to a suspected diagnosis, such as cancer [6].

Diagnostic delay and its correlation to mortality in cancer is still a matter of discussion, especially to what extent fewer time delays would decrease mortality in cancer [4,7,8]. A Danish study after CPPs implementation in Denmark, showed that the survival rate rose and the excess mortality decreased for seven different types of cancer but the results were only statistically significant for lung and gynaecological cancer [9]. This study and others illustrate the heterogeneity of cancer, suggesting that reduced diagnostic delay may have a large impact on survival in certain cancers but a lower to no effect on others [7,10].

Sweden has a high cancer survivability rate but suffers from problems of drawn-out waiting times in some fields of cancer care, along with large regional differences and overall a high proportion of discontented patients [11]. To improve this, Sweden followed Denmark’s footsteps and started implementing their own CPP programme in 2015, called ‘standariserade vårdförlopp i cancervården’ in Swedish [11]. In November 2020, 31 different CPPs were implemented in Sweden. The suspicion and treatment of cancer can be divided into four parts: entry, investigation, treatment and follow-up. Currently in Sweden, CPPs cover a description of the entry and investigation parts. The entry part comprises both suspicion and well-founded suspicion of cancer. The investigation part starts with the first appointment with a hospital/organ specialist, and may continue with further diagnostics, a multidisciplinary conference and eventual decisions regarding the start of treatment. Today, in Sweden, a CPP ends with the start of treatment of cancer in the patient, or with negative findings ruling out the cancer suspected according to the medical referral of a specific CPP [11].

The role of GPs in the detection of cancer is underlined because most cancer patients are diagnosed after presenting symptoms and findings in a primary care unit [1,10,12,13]. For GPs, the continuity of care and the prior knowledge of a patient over time, are believed to be crucial for an early suspicion of a cancer diagnosis [6]. A challenge for GPs is that the positive predictive power of a warning sign or symptom for cancer is lower in primary care than in secondary care, which is explained by the spectrum effect [14–16].

Disbeliefs against the benefits with systems such as Swedish CPPs are that the complexity of the diagnostic process is overlooked and that standardization increases the risk of over testing and crowding-out effects. [6,17–19].

There are two important aspects for the future realization of the goals set up for CPPs. One necessity is that the different CPPs are well structured and organized for the effective detection and investigation of cancer. The other aspect is that the knowledge of these pathways properly reaches out to and is applied by the working physicians. Therefore, the main aim of this study was to test knowledge regarding the initiating (entry) procedure of CPPs among GPs in one group sorted out from other physicians. Second, there was also an interest in determining physicians’ opinions of and self-estimated knowledge about CPPs.

## Material and methods

This study started with the construction of a pilot questionnaire in Swedish about the Swedish system for initiating a CPP. The pilot questionnaire were tested by an expert group and thereafter modified to its final version (see the questionnaire translated into English in Supplemental Material I). Respondents of the study were physicians from two different regions (Örebro County and Värmland County) in Sweden. They were asked about their age, medical specialty, professional title, working years as physicians, and their estimation of the total number of CPPs they had initiated (Tables 1). Approximately half of the recruited respondents were informed about and asked to participate in the study during a local assembly among physicians from the two regions of Värmland and Örebro in January 2019. The other physicians were recruited from November 2018 throughout January 2019 at the University Hospital in Örebro as well as two different primary care units within the region of Örebro. These respondents were recruited in personal areas such as lunch or conference rooms. Most of these questionnaires were handed out in person and collected immediately after they were filled out. A small number, less than 10%, send in their answers later by post. Medical students were informed about the study and were asked to participate and fill out the questionnaire during a lecture at Örebro University in November 2018.

**Table 1.** Background data on participants in the survey.

	Junior doctors/ students	Resident physicians	Other specialists	GPs	Total
Participants	34	33	29	59	155
Age	25 (23–26)	34 (32–35)	47 (42–51)	51 (48–53)	41 (38–43)
Years working	0.4 (0.2–0.6)	4.9 (4.0–5.8)	19 (16–21)	22 (20–25)	13 (11–15)
CPPs initiated	2.4 (0.5–4.3)	18 (13–23)	15 (5–25)	24 (19–29)	16 (13–19)

Mean values for the different groups of physicians based on their age, years working, and number of CPPs initiated. The number of participants for each group in ‘Total’ column is given their exact values, and other values in the table are rounded off to two significant figures. 95% confidence intervals in parenthesis.

**Table 2.** The results for the 10 MCQs for different groups of physicians were divided into three categories with 0–3, 4–5 and 6–10 correct answers.

Correct answers	Junior doctors/ students	Resident physicians	Other specialists	GPs	Total
0–3	65% (22)	39% (13)	62% (18)	31% (18)	46% (71)
4–5	32% (11)	36% (12)	31% (9)	39% (23)	36% (55)
6–10	3% (1)	24% (8)	7% (2)	31% (18)	19% (29)
Mean of correct answers	2.6	4.2	3.2	4.5	3.8
CI of correct answers	2.0–3.1	3.4–4.9	2.5–3.8	4.0–5.1	3.5–4.1
Range: Min–Max	0–6	1–9	0–7	1–9	0–9

The relative number of participants in each group is given in percentages (%) and absolute values in parentheses. The group junior doctors include medical students and doctors before entering a residency, and the other specialists contain specialists and chief physicians who are not GPs. Mean, range and 95% confidence intervals show the results and distribution in the different groups. Values in percentages are rounded off to an integer percent. Other values in the table are rounded off to two significant figures.

**Table 3.** The table displays the responding physicians’ answers to the questions ‘Can CPPs reduce mortality and morbidity in cancer?’ and ‘Do you think you have sufficient knowledge regarding the initiating procedure of CPPs?’.

	Resident physicians	Other specialists	GPs
Reduce mortality/morbidity?			
No	10% (3)	20% (4)	4% (2)
Yes	71% (22)	60% (12)	77% (37)
Don’t know	19% (6)	20% (4)	19% (9)
Sufficient knowledge?			
No	48% (15)	85% (17)	52% (27)
Yes	29% (9)	10% (2)	38% (20)
Do not know	23% (7)	5% (1)	10% (5)

The answers are presented for each group of physicians in percent and in integer numbers within the parentheses. Total numbers (the ‘Total’ column), depending on physicians’ answers on each row (no, yes and do not know) for all physicians are given with their corresponding mean correct answers in the previous MCQs and 95% confidence intervals in the last columns to the right. Values in percentages are rounded off to an integer percent. Other values in the table are rounded off to two significant figures.

From 10 different cancer types and their corresponding CPP, 1 question each was constructed. MCQs 1–5 included common types of cancer (prostate cancer, breast cancer, lung cancer, colorectal cancer, and cancer in the bladder and upper urinary tracts). MCQs 6–10 included uncommon types of cancer (acute leukaemia, vulvar cancer, penis cancer, anal cancer, and neuroendocrine tumors in the abdomen including cancer in the adrenals). The questions were posed as MCQs with three different choices and one correct answer for each question. These MCQs were combined in a questionnaire; along with two additional questions, one asking the physicians whether they thought CPPs would lead to a decrease in cancer mortality and morbidity, and the second asking them to evaluate their self-estimated knowledge regarding CPPs.

At the time of the construction of this questionnaire, the questions were designed based on the CPP document version 5.0. By December 17, 2018, the document was updated to version 6.0, which brought changes to several CPPs. Only the colorectal cancer question was affected by this. By version 6.0, changes to bowel habits no longer qualified as criteria for well-founded suspicion, and therefore, the answer to that question was not correct (see Supplemental Materials I and II, question 4). Since all MCQ were formed from version 5.0, they were still corrected in accordance to that version.

About 240 questionnaires were handed out to physicians and medical students and 163 of those agreed to participate in completing the survey. GPs comprised the largest group (*n* = 59). The other specialists (*n* = 29) mainly consisted of specialists/subspecialists within internal medicine (*n* = 17) or surgery (*n* = 10) (Table 1). The group of junior doctors comprised medical students, physicians who had not yet obtained a medical license and physicians with a medical license but had not begun residency (Table 1).

Seven of the 163 participants were excluded due to answering fewer than 8 out of 10 MCQs. One additional participant was excluded because there was no answer to the background questions. After these exclusions, the number of participants left for descriptive statistics was 155, giving a response rate of 65%.

### Analysis

For the analysis of the confidence interval, a two-sided t-test was used. Data analysis was performed using SPSS version 25 (IBM Corp. Released 2017. IBM SPSS Statistics for Windows, Version 25.0. Armonk, NY: IBM Corp).

## Results

The 155 participants in the survey had a mean age of 41 years, 13 years of experience as a physician and had initiated 16 CPPs (Table 1). The mean age for GPs (51 years), other specialists (47 years) and resident physicians (34 years) within our study is comparable to the average age of specialists (49 years) and resident physicians (35 years) in Sweden according to official data from 2016 from the National Board of Health and Welfare. The mean score for the MCQs across all tested physicians was 3.8 out of a maximal score of 10 (Table 2). GPs had the highest mean of correct answers 4.5, compared to resident physicians 4.2, chief physicians and other specialists 3.2, and the group with junior doctors (including medical students) 2.6 (Table 2). The portion of GPs with more than 50% correct answers to the survey was approximately four times larger than the portion of other specialists with more than 50% correct answers (Table 2). In total (of all 155 participants), 46% had 0–3 correct answers, 36% had 4–5 correct answers, and 19% had 6–10 correct answers (Table 2).

When asked if the physicians thought CPPs would lead to a decrease in mortality and morbidity in cancer, 70% answered yes. 8% answered no, and 22% answered ‘do not know’ (Table 3). This judgement did not correlate to any significant difference in the number of correct answers on the survey (Table 3). When asked if the physicians thought they had sufficient knowledge regarding the procedure of CPPs 63% answered no, 26% answered yes, and 10% answered ‘do not know’ (Table 3). The mean number of correctly answered questions was significantly higher among those who thought they had sufficient knowledge 4.9, than among those who did not feel that they had sufficient knowledge 3.4 (Table 3). A minority in all groups thought they had sufficient knowledge about CPPs, with GPs 39%, resident physicians 29%, and other specialists 10%, on par with junior doctors 10% (Table 3).

## Discussion

### Statement of principal findings

This survey study showed that Swedish physicians’ knowledge of CPPs was low, yet GPs performed better than other physicians did. Overall, a clear majority of physicians rated their knowledge as lacking regarding the procedure of CPPs. Regardless, most physicians believed that the whole system, i.e. both the entry and investigation parts of CPPs, promote a lower mortality and morbidity in cancer.

### Strengths and weaknesses

The 10 knowledge-based MCQs were constructed to be quite difficult such that it would be possible to differentiate between the knowledge among groups of physicians. It proved to be so difficult that as many as 46% (three or fewer correct answers) performed worse than if had they answered randomly (3.33 correct answers) (Table 2). More than one answer on each question could give rise to the suspicion of cancer, but only one answer (the correct one) was derived from the corresponding CPP. Because of this, the general expertise of when to suspect cancer was not tested in this paper; merely the know-how about the specific rules and guidelines for the suspicion of cancer according to the CPPs was tested. One could also argue about the necessity for physicians to know each specific symptom or finding for different CPPs since these are easy to access at different websites. The generalization of findings in this study could also be questioned because geographic spread was limited to two regions in Sweden (one with a university hospital). The main cities in these two regions represent the 6th and 20th largest cities in Sweden. However, CPPs is a national system with small differences in application and organization of the entry part between regions in Sweden. Another limitation to this study is that only a few background questions were posed about participants and therefore other variables or confounding factors could be of importance for interpretation of the results. Nonresponse analysis was not used in those who declined to answer this survey.

### Meaning of the study: possible mechanisms and implications for clinicians or policy makers

Early detection of cancer in patients is believed to be important for the outcome of cancer treatments. The newly launched system with CPPs presents guidelines for symptoms and findings, which aims to improve the detection of cancer at an early stage and direct patients to the right investigation.

Those physicians who believed they had insufficient knowledge about CPPs also performed significantly worse than those who thought their knowledge was sufficient. However, as a group, the more confident physicians had a mean of 4.9 correct answers, which is slightly below 50% correct answers. When asked about their self-estimated knowledge the vast majority of physicians felt that it was not sufficient. This pinpoints an overall meagre knowledge about CPPs, even though a majority of physicians believed that the whole system (entry and investigation) with CPPs would lead to a decrease in cancer mortality and morbidity. Since several of the findings and symptoms of cancer are included in different CPPs it is not always clear which CPP to select. One study from Denmark identified this as a medical risk by showing that patients who were cleared from cancer in one CPP had a significantly higher risk of presenting with another cancer within 6 months [20]. This study highlights possible improvements in current CPPs, for example, a finding of gastrointestinal bleeding is currently included in three different CPPs and macroscopic haematuria is included in two, both for suspected kidney cancer and for suspected urinary and bladder cancer. The risk of missing a cancer in such a system would probably decrease if bleeding from the gastrointestinal tract or macroscopic haematuria were treated as suspected cancer in all relevant organs after referral for a CPP based on the presented finding in the patient. These are examples of symptoms and findings where a more general approach for the entry part could simplify the selection of CPP for physicians.

As a final conclusion, this article notes that more work is required in teaching and training physicians about the system with CPPs, in order for it to be more timely implemented for cancer detection.

## Supplementary Material

Supplemental Material
