# Supplementary material for: Knowledge of and support for cancer patient pathways among general practitioners and other physicians – a study from Sweden
Source: Scand J Prim Health Care. 2021 Feb 8;39(1):17–22. doi: 10.1080/02813432.2021.1880074 (PMC7971341; doi:10.1080/02813432.2021.1880074)
Supplement: Supplemental Material [file IPRI_A_1880074_SM3184.docx]

**Supplemental material I**

**English version of the survey**

**Knowledge in and support for cancer patient pathways (CPPs) among GPs and other physicians in Sweden**

| Age: |  | Medical speciality: |  |
| --- | --- | --- | --- |
| Work title: |  | Years spent as a working physician: |  |

| Estimate the amount of CPPs you have initiated: ______________________________________________________ |
| --- |

The following questions are founded from the document “Standardiserade vårdförlopp – förkortade versioner för primärvård” by “Regionala cancercentrum i samverkan”. The questions are shaped after which finds give rise to suspicion/well-founded suspicion for 10 different types of cancer, 5 common and 5 uncommon (estimated from the number of diagnosed cases per year). Please cross the alternative you think applies to each question. There is only ONE correct alternative per question.

1. Which of the following answers with findings shall according to CPPs lead to a well-founded suspicion of breast cancer?

- 1. Pain in breast tissue in a patient with at least one first-degree relative diagnosed with breast cancer before the age of 60.
- X. Eczema on one of the nipples.
- 2. Newly discovered hyperprolactinemia in a patient with a confirmed BRCA mutation.

2.

- .

3. Which of the following answers with finding shall according to CPPs lead to a suspicion of prostate cancer?

- 1. Men older than 40 years with a confirmed BRCA2 mutation.
- X. Urinary problems among elderly men with an expected survival of at least more than 10 years.
- 2. LUTS problematic among grown men regardless of age.

4. Which of the following answers with findings shall according to CPPs lead to an immediate referral, without any other investigation, in cases of well-founded suspicion of colorectal cancer?

- 1. Positive faeces-Hb x 3 in a patient with dark stool and stable bowel habits.
- X. Newly discovered anaemia with blood in the stool in a patient above the age of 50.
- 2. A change in otherwise stable bowel habits for more than 4 weeks, without any other explanation, in patients above the age of 40.

5. Which of the following answers with findings shall according to CPPs lead to a well-founded suspicion of cancer in the bladder and upper urinary tracts?

- 1. Individuals above the age of 40 with repeated microscopic haematuria and micturition difficulties.
- X. Individuals above the age of 50 with macroscopic haematuria on a single occasion, under simultaneous treatment with anticoagulants.
- 2. Individuals above the age of 50 with micturition difficulties without any other explanation.

6. Which of the following answers with findings (without any other obvious reason) shall according to CPPs lead to a suspicion of acute leukaemia?

- 1. One or more enlarged lymph nodes over 2 cm.
- X. New onset tiredness and general malaise.
- 2. Skeletal pains from the hip, back or legs.

7. Which of the following answers with findings (without any obvious reason) shall according to CPPs, lead to a suspicion of vulvar cancer among women above the age of 40, or women with an immune deficiency regardless of age?

- 1. Prolonged pain or itching which does not get relieved by treatment.
- X. A woman with a first-degree relative diagnosed with vulvar cancer, as well as a mutation in the CPP2 gene.
- 2. Dyspareunia without any other explanation.

8. Which of the following answers shall according to the CPPs lead to a well-founded suspicion of penis cancer? (NOTE that the two wrong answers give reason to suspect penis cancer, but not according to a well-founded suspicion)

- 1. Pain from the penis as well as an enlarged lymph node in the groin.
- X. A wound on the penis that will not heal in a man with an age above 50.
- 2. An indistinct lump on the penis in a man at an age above 40.

9. Which of the following answers with findings shall according to CPPs lead to a well-founded suspicion of anal cancer?

- 1. A patient with at least two of the following risk factors for anal cancer: A high consumption of red meat, smoking, a first-degree relative with known anal cancer or males who have sex with males (MSM).
- X. New onset or altered anal symptoms, without any other explanation, among males who have sex with males (MSM).
- 2. Anaemia in a patient associated with any known risk group: HIV-positive, males who have sex with males, immunosuppressed persons, or a person who has or has had HPV-related genital in situ or invasive neoplasia.

10. Which of the following answers with findings (without any obvious explanation) shall according to CPPs lead to a suspicion of neuroendocrine abdominal tumours including adrenal cancer?

- 1. Lasting diarrhoea for more than three months.
- X. Recurring HP-positive gastric ulcer.
- 2. P-APT-time over 33 s (normal reference interval 26-33s).

Do you think CPPs, with support for when to suspect / well-founded suspicion of cancer, will lead to a reduced mortality and morbidity in cancer (yes, no, don’t know)? Please discuss your answer below.

Do you personally think that you have sufficient knowledge regarding the procedure of CPPs (yes/no/do not know)? Please discuss your answer below.

**Supplemental material II**

**Table showing the distribution of answers for each MCQ alternative among the different groups of physicians and among all the groups combined. The proportion (%) of how physicians have answered is given after each choice (1, X or 2).**

| **Correct answer** | Junior doctors/Intern physician/Students n= 34 | Resident physician n= 33 | Chief physicians/ specialists n= 29 | GPs= 59 | Total  N= 155 |
| --- | --- | --- | --- | --- | --- |
| 1. X | 1. 58.8  X. 8.8  2. 32.4 | 1. 30.3  X. 45.5  2. 24.2 | 1. 37.9  X. 37.9  2. 24.1 | 1. 25.4  X. 47.5  2. 27.1 | 1. 36.1  X. 36.8  2. 27.1 |
| 2. 2 | 1. 64.7  X. 14.7  2. 20.6 | 1. 51.6  X. 19.4  2. 29.0 | 1. 58.6  X. 31.0  2. 10.3 | 1. 50.8  X. 30.5  2. 18.6 | 1. 55.6  X. 24.8  2. 19.6 |
| 3. X | 1. 26.5  X. 38.2  2. 35.3 | 1. 25.0  X. 37.5  2. 37.5 | 1. 41.4  X. 31.0  2. 27.6 | 1. 31.0  X. 34.5  2. 34.5 | 1. 30.7  X. 35.3  2. 34.0 |
| 4. 2 | 1. 14.7  X. 47.1  2. 38.2 | 1. 0.0  X. 53.1  2. 46.9 | 1. 17.2  X. 62.1  2. 20.7 | 1. 3.4  X. 52.5  2. 44.1 | 1. 7.8  X. 53.2  2. 39.0 |
| 5. X | 1. 41.2  X. 32.4  2. 26.5 | 1. 15.6  X. 75.0  2. 9.4 | 1. 62.1  X. 31.0  2. 6.9 | 1. 11.9  X. 86.4  2. 1.7 | 1. 28.6  X. 61.7  2. 9.7 |
| 6. X | 1. 50.0  X. 23.5  2. 26.5 | 1. 59.4  X. 18.8  2. 21.9 | 1. 69.0  X. 27.6  2. 3.4 | 1. 70.7  X. 15.5  2. 13.8 | 1. 63.4  X. 20.3  2. 16.3 |
| 7. 1 | 1. 41.2  X. 38.2  2. 20.6 | 1. 45.5  X. 21.2  2. 33.3 | 1. 51.7  X. 31.0  2. 17.2 | 1. 64.4  X. 30.5  2. 5.1 | 1. 52.9  X. 30.3  2. 16.8 |
| 8. 1 | 1. 32.4  X. 44.1  2. 23.5 | 1. 12.1  X. 84.8  2. 3.0 | 1. 3.4  X. 69.0  2. 27.6 | 1. 6.9  X. 84.5  2. 8.6 | 1. 13.0  X. 44.4  2. 38.6 |
| 9. X | 1. 38.2  X. 17.6  2. 44.1 | 1. 9.1  X. 45.5  2. 45.5 | 1. 13.8  X. 35.5  2. 51.7 | 1. 10.5  X. 64.9  2. 24.6 | 1. 17.0  X. 44.4  2. 38.6 |
| 10. 1 | 1. 41.5  X. 23.5  2. 35.3 | 1. 68.8  X. 28.1  2. 3.1 | 1. 69.0  X. 20.7  2. 10.3 | 1. 77.2  X. 12.3  2. 10.5 | 1. 65.8  X. 19.7  2. 14.5 |
